# A Proteomic Analysis of Human Follicular Fluid: Comparison between Younger and Older Women with Normal FSH Levels

**DOI:** 10.3390/ijms151017518

**Published:** 2014-09-29

**Authors:** Mahmoud Hashemitabar, Maryam Bahmanzadeh, Ali Mostafaie, Mahmoud Orazizadeh, Marzieh Farimani, Roshan Nikbakht

**Affiliations:** 1 Cellular and Molecular Research Center, Faculty of Medicine, Ahvaz Jundishapur University of Medical Sciences, Ahvaz 1579461357, Iran; E-Mails: hashemi-m@ajums.ac.ir (M.H.); m_orazizadeh@yahoo.com (M.O.); 2 Medical Biology Research Center, Kermanshah University of Medical Sciences, Kermanshah 6714415185, Iran; E-Mail: amostafaie@kums.ac.ir; 3 Endometr and Endometriosis Research Center, Hamedan University of Medical Sciences, Hamedan 6517789971, Iran; E-Mail: dr_Farimani@yahoo.com; 4 Fertility and Infertility & Perinatology Research Center, Ahvaz Jundishapur University of Medical Sciences, Ahvaz 6193673166, Iran; E-Mail: Rosnikba@yahoo.com

**Keywords:** follicular fluid, reproductive aging, proteomic analysis, MALDI-TOF-TOF, two-dimensional gel electrophoresis

## Abstract

The follicular fluid (FF) is produced during folliculogenesis and contains a variety of proteins that play important roles in follicle development and oocyte maturation. Age-related infertility is usually considered as a problem that can be solved by assisted reproduction technology. Therefore, the identiﬁcation of novel biomarkers that are linked to reproductive aging is the subject of this study. FF was obtained from healthy younger (20–32 years old) and older (38–42 years old) women undergoing intracytoplasmic sperm injection (ICSI) due to male factor infertility. The FF was analyzed by two-dimensional gel electrophoresis (2-DE). The power of two-dimensional gel electrophoresis and the identiﬁcation of proteins were exploited using matrix-assisted laser desorption-ionization time-of-flight/time-of-flight (MALDI-TOF-TOF) mass spectrometry. Twenty three protein spots showed reproducible and significant changes in the aged compared to the young group. Of these, 19 protein spots could be identified using MALDI-TOF-TOF-MS. As a result of MASCOT search, five unique downregulated proteins were identified in the older group. These were identified as serotransferrin, hemopexin precursor, complement C3, C4 and kininogen. A number of protein markers were found that may help develop diagnostic methods of infertility.

## 1. Introduction

The follicular fluid (FF) is produced during folliculogenesis and contains a variety of proteins that play important roles in follicle development and oocyte maturation. FF results from the transfer of blood plasma components and the secretory activity of the oocyte, granulosa and thecal cells. It contains over 200 different proteins that reflect the stage of oocyte development and the degree of follicle maturation [1,2,3]. Several studies explained the presence of gonadotropins and steroids in the FF as the markers for oocyte maturation [4,5,6]. The other factors, such as vascular endothelial growth factor (VEGF ) [7,8,9], inhibin A and B[8], anti-Müllerian hormone [10], lactoferrin [11], insulin-like growth factor-II (IGF-II) [12], hyaluronan [13], nitric oxide [9,14], leptin [15,16], 25-OH vitamin D, glucose [17], bone morphogenetic proteins (BMPs) [18,19] and interleukin 8 [20], correlate with oocyte maturation and folliculogenesis. This microenvironment may be altered by conditions, such as endometriosis [21,22,23], polycystic ovary syndrome [24] and reproductive aging [25,26,27]. Reproductive aging is marked by the decline in ovarian response, oocyte number [1,28,29,30,31] and quality [30]. Female age, quality and quantity of the retrieved oocytes are the major factors affecting *in vitro* fertilization (IVF) outcomes. It has been previously shown that altered FF composition is associated with diminished reproductive capacity [18,31]. Some of the FF markers could be correlated to reproductive aging, such as interleukin-8 [20] GDF-9 and TGF-β1 [32], inhibin [33], anti-Müllerian hormone [10] and bone morphogenetic protein 15 [18]. Therefore, proteome analysis of FF might be helpful in evaluating oocyte quality and identifying predictive markers for oocyte developmental potential prior to fertilization and the success in assisted reproductive technology (ART) [1,20,34]. Two-dimensional electrophoresis (2-DE) was used by Spitzer *et al.* (1996), for the first time, to compare protein patterns of mature and immature human follicles [35].

The first attempts to elucidate the FF proteome led to the detection of new proteins, including thioredoxin peroxidase 1(TDPX1), transthyretin (TTR), retinol-binding protein (RBP) [36], hormone sensitive lipase (HSL), unnamed protein product 1 (UPP1), unnamed protein product 2 (UPP2) and apolipoprotein A-IV precursor [37]. Subsequently, the FF proteome map was compared with serum in the superovulatory cycle [38]. Several studies that have applied proteomic analyses to assess human FF have provided information on the pathophysiology of conditions, such as recurrent spontaneous abortion [39], polycystic ovary syndrome [24], endometriosis [23], ovarian hyperstimulation syndrome [40] and failure to become pregnant after IVF [41]. Based on the physiological interdependence between FF and oocytes, we hypothesized that female age-related reproductive decline may be correlated with deleterious alterations in FF physiology. The proper assessment of ovarian aging at an early stage seems to be crucial for counseling patients about their chances for pregnancy, either spontaneously or during fertility therapy [42]. Meta-analysis and systematic reviews have failed to identify any combination of specificity and sensitivity for basal FSH as a test of poor response or prediction of non-pregnancy. In regularly-cycling women, only extremely elevated results are useful in predicting poor response to ovarian stimulation, so this will be barely helpful for screening test and counseling purposes [43,44]. It is understood that ovarian aging begins several years before any elevation in FSH levels is noted, and hence, a normal test cannot rule out a poor ovarian response in some women [45].

We believe that the component differences can be characterized by FF protein profile during ovulation. The purpose of this study, therefore, was to assess the differences in the protein profile of FF between older and younger reproductive women undergoing intracytoplasmic sperm injection (ICSI).

## 2. Results

### 2.1. Clinical Characteristics of Patients

There were no differences in FSH and BMI among older and younger patients (Table 1). The mean ages were 25.6 ± 3.2 and 39.6 ± 2.1 for the younger and older groups, respectively. The mean number of oocytes from both groups is listed in Table 1.

### 2.2. Number of Retrieved Mature Oocytes

The number of retrieved metaphase II oocytes was significantly lower in the older group (*p* < 0.05) (Table 1).

**Table 1:** Clinical characteristics of patients.

Characteristics	Younger	Older
Mean age (year)	25.6 ± 3.2	39.6 ± 2.1 *
BMI (kg/m^2^)	26.8 ± 2.6	28.6 ± 1.5
Basal FSH (mIU/mL)	6.1 ± 1.2	6.9 ± 1.9
No. of MII oocytes	9 ± 4.4	3.3 ± 4.1 *
Serum Transferrin (mg/dL)	302.5 ± 38.1	251.5 ± 38.7 *

Values are presented as the mean ± SD; BMI, body mass index; MII, metaphase II; * *p* < 0.05.

### 2.3. Albumin/IgG Depletion

**Figure 1:**
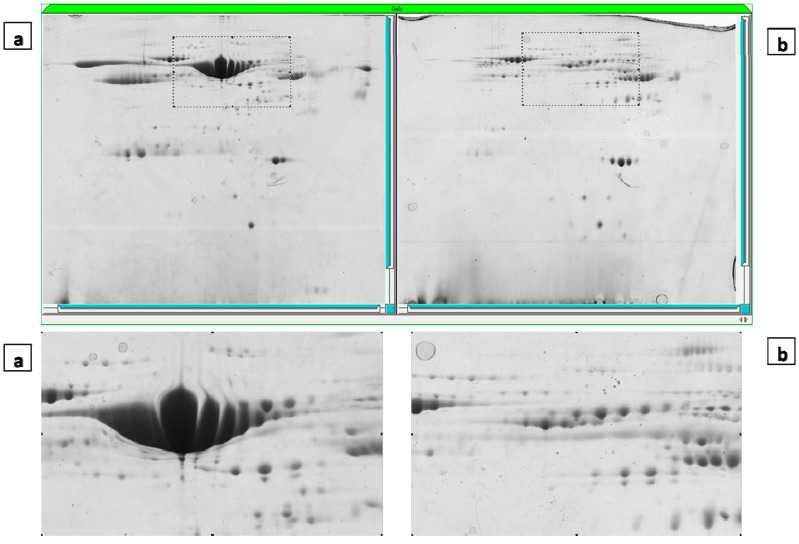
Two-dimensional gel electrophoresis map of crude (**a**) and albumin/IgG-depleted follicular fluid (**b**) (Coomassie blue staining).

In order to remove Albumin and IgG from FF, a Qproteome Albumin/IgG Depletion Plate was used to visualize and identify some low abundance proteins. Thus, the lowest percentage of depletion efficiency was confirmed by 2-DE (Figure 1).

### 2.4. Gel Imaging

**Figure 2:**
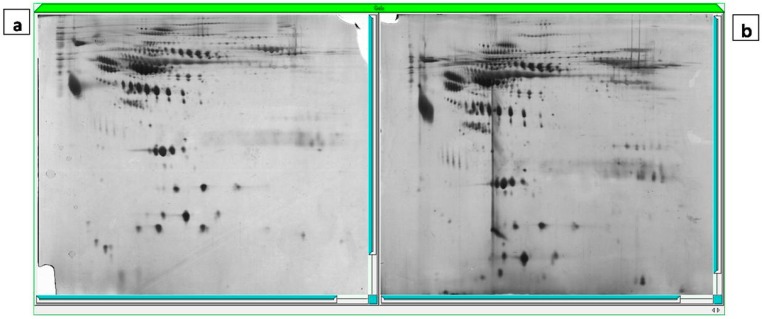
Two-dimensional gel electrophoresis map of young (**a**) and aged groups (**b**) (silver staining).

The 2D proteins patterns were almost similar between the two groups (Figure 2). The mean number of spots detected per gel was 255. Twenty three protein spots with significant differences in expression levels were observed between the two groups.

### 2.5. The Mass Spectrometry Assessment and MASCOT Report

Twenty three protein spots (which underwent changes in expression level) were excised from the gels and were analyzed by MALDI-TOF-TOF. Of these, 19 protein spots could be identified using MALDI-TOF-TOF-MS. five unique proteins and the MASCOT supporting identification data are listed in Table 2. Sequence similarity is available as an NCBI BLAST search of accession number.

**Figure 3:**
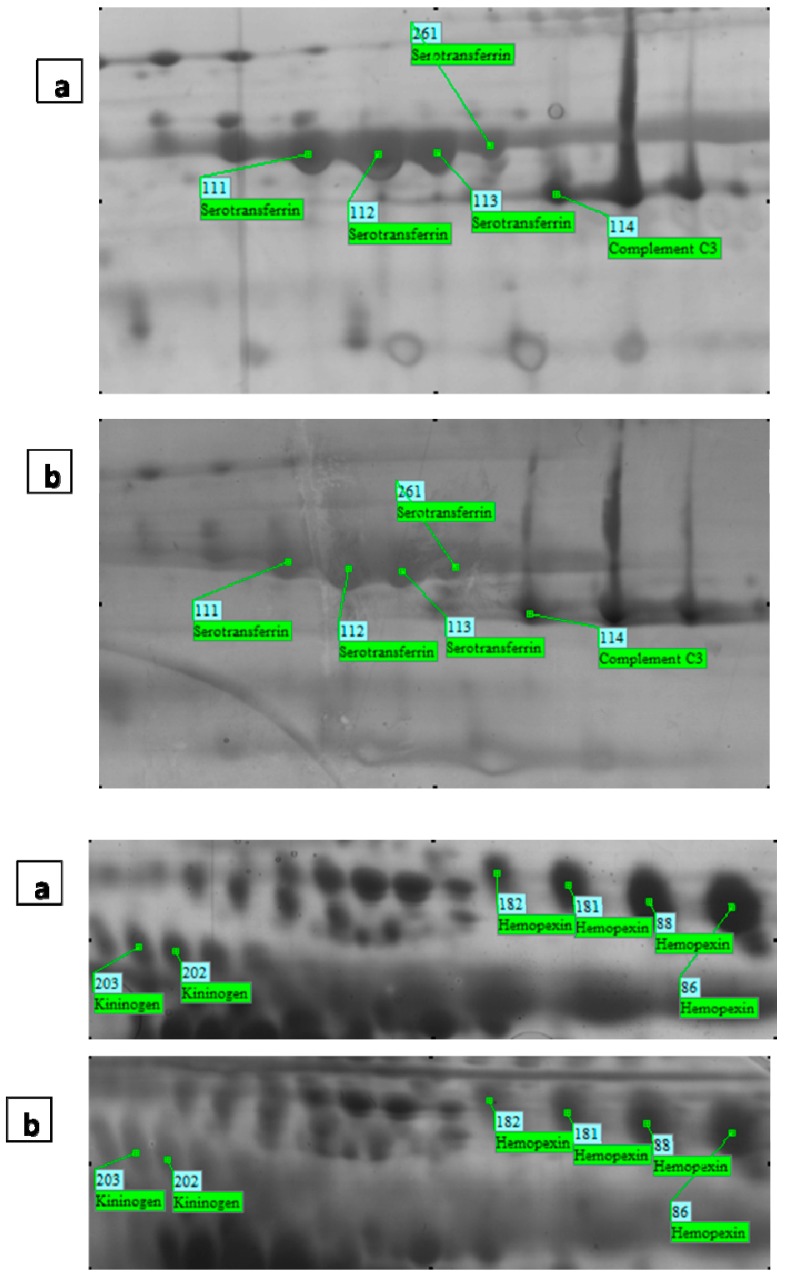
Magnified 2-DE maps of representative spots with differential expression between the young group (**a**) and the aged group (**b**).

The proteins included hemopexin (spots 75, 86, 88, 90, 91, 181, 182), kininogen-1 (spots 202, 203), STF (spots 108, 111, 112, 113, 261, 254), complement C3 (spots 114) and complement C4 (spots 199, 200, 285) (Figure 3). Differentially-expressed protein spots were observed at significantly decreased levels in the FF of older patients.

**Table 2:** Proteins identified using MALDI-TOF-TOF mass spectrometry.

Spot ID ^a^	MASCOT Search Results						Accession Number (SWISS-PROT)	Protein Name	Fold Change ^f^	Peptide
	Molecular Mass (kDa)	pI ^b^	No. of Matched Peptides	Sequence Coverage ^c^ (%)	Score ^d^	Accession Number ^e^				
75	52,385	6.55	6	18	396	IPI00022488	P02790	Hemopexin	−1.59	K.GSFPWQAK.M R.VGYVSGWGR.N R.HYEGSTVPEK.K K.SCAVAEYGVYVK.V K.YVMLPVADQDQCIR.H K.YVMLPVADQDQCIR.H
86	52,385	6.55	5	17	539	IPI00022488	P02790	Hemopexin	−1.51	K.NFPSPVDAAFR.Q R.YYCFQGNQFLR.F R.GECQAEGVLFFQGDR.E K.LLQDEFPGIPSPLDAAVECHR.G K.EVGTPHGIILDSVDAAFICPGSSR.L
88	52,385	6.55	3	8	256	IPI00022488	P02790	Hemopexin	−1.53	K.NFPSPVDAAFR.Q R.GECQAEGVLFFQGDR.E R.YYCFQGNQFLR.F
90	52,385	6.55	5	14	473	IPI00022488	P02790	Hemopexin	−1.67	K.NFPSPVDAAFR.Q R.FDPVRGEVPPR.Y R.YYCFQGNQFLR.F R.GECQAEGVLFFQGDR.E K.LLQDEFPGIPSPLDAAVECHR.G
91	52,385	6.55	4	12	358	IPI00022488	P02790	Hemopexin	−1.51	K.NFPSPVDAAFR.Q K.LLQDEFPGIPSPLDAAVECHR.G R.GECQAEGVLFFQGDR.E R.YYCFQGNQFLR.F
181	52,385	6.55	4	12	382	IPI00022488	P02790	Hemopexin	−1.51	K.NFPSPVDAAFR.Q K.LLQDEFPGIPSPLDAAVECHR.G R.GECQAEGVLFFQGDR.E R.YYCFQGNQFLR.F
182	52,385	6.55	3	8	181	IPI00022488	P02790	Hemopexin	1.67	K.NFPSPVDAAFR.Q R.GECQAEGVLFFQGDR.E R.YYCFQGNQFLR.F
202	48,936	6.29	3	7	224	IPI00215894	P01042	Kininogen-1	−2.2	K.YNSQNQSNNQFVLYR.I R.QVVAGLNFR.I K.EETTSHLR.S
203	48,936	6.29	7	14	427	IPI00215894	P01042	Kininogen-1	−1.8	K.YNSQNQSNNQFVLYR.I K.TVGSDTFYSFK.Y R.QVVAGLNFR.I K.KYFIDFVAR.E K.YFIDFVAR.E K.RPPGFSPFR.S K.EETTSHLR.S
108	79,294	6.81	10	16	731	IPI00022463	P02787	Serotransferrin	−2.03	R.APNHAVVTR.K K.ASYLDCIR.A K.WCALSHHER.L K.EGYYGYTGAFR.C K.DYELLCLDGTR.K K.MYLGYEYVTAIR.N K.CSTSSLLEACTFR.R R.DQYELLCLDNTR.K R.KPVEEYANCHLAR.A K.DCHLAQVPSHTVVAR.S
111	79,294	6.81	10	15	846	IPI00022463	P02787	Serotransferrin	−2.1	K.DSGFQMNQLR.G R.DQYELLCLDNTR.K K.DCHLAQVPSHTVVAR.S K.MYLGYEYVTAIR.N K.EGYYGYTGAFR.C K.NLNEKDYELLCLDGTR.K K.DYELLCLDGTR.K R.KPVEEYANCHLAR.A R.APNHAVVTR.K K.CSTSSLLEACTFR.R
112	79,294	6.81	10	16	752	IPI00022463	P02787	Serotransferrin	−2.01	R.APNHAVVTR.K K.ASYLDCIR.A K.WCALSHHER.L K.EGYYGYTGAFR.C K.DYELLCLDGTR.K K.MYLGYEYVTAIR.N K.CSTSSLLEACTFR.R R.DQYELLCLDNTR.K R.KPVEEYANCHLAR.A K.DCHLAQVPSHTVVAR.S
113	79,294	6.81	10	16	787	IPI00022463	P02787	Serotransferrin	−1.51	K.ASYLDCIR.A R.DQYELLCLDNTR.K K.DCHLAQVPSHTVVAR.S K.MYLGYEYVTAIR.N K.WCALSHHER.L K.EGYYGYTGAFR.C K.DYELLCLDGTR.K R.KPVEEYANCHLAR.A R.APNHAVVTR.K K.CSTSSLLEACTFR.R
261	79,294	6.81	9	14	689	IPI00022463	P02787	Serotransferrin	−1.52	K.ASYLDCIR.A R.DQYELLCLDNTR.K K.DCHLAQVPSHTVVAR.S K.MYLGYEYVTAIR.N K.WCALSHHER.L K.EGYYGYTGAFR.C K.DYELLCLDGTR.K R.KPVEEYANCHLAR.A K.CSTSSLLEACTFR.R
254	79,294	6.81	10	16	724	IPI00022463	P02787	Serotransferrin	−2.06	K.ASYLDCIR.A R.DQYELLCLDNTR.K K.DCHLAQVPSHTVVAR.S K.MYLGYEYVTAIR.N K.WCALSHHER.L K.EGYYGYTGAFR.C K.DYELLCLDGTR.K R.KPVEEYANCHLAR.A R.APNHAVVTR.K K.CSTSSLLEACTFR.R
114	188,569	6.02	3	2	222	IPI00783987	P01024	Complement C3	−1.6	K.TIYTPGSTVLYR.I K.KVEGTAFVIFGIQDGEQR.I R.IPIEDGSGEVVLSR.K
199	194,247	6.65	2	1	91	IPI00032258	P01028	Complement C4-A	−2.2	R.EELVYELNPLDHR.G R.QGSFQGGFR.S
200	194,170	6.89	4	2	219	IPI00418163	P0C0L5.2	Complement C4-B preproprotein	−2.19	R.EFHLHLR.L R.EELVYELNPLDHR.G K.AEMADQAAAWLTR.Q R.QGSFQGGFR.S
285	194,247	6.65	2	1	80	IPI00032258	P01028	Complement C4-A	−1.78	K.GLCVATPVQLR.V R.EELVYELNPLDHR.G

^a^ Spots are numbered according to the 2-DE gel; ^b^ isoelectric point; ^c^ percentage of the protein sequence covered by identified peptides; ^d^ the protein score is the sum of all of the ion scores of all of the peptides; ^e^ the MASCOT results of MALDI-TOF-TOF; ^f^ the fold changes in downregulated proteins are shown as negative values.

### 2.6. Serum Transferrin Level

To provide insights into the effect of aging on the serum transferrin level, we measured this protein. The mean serum transferrin levels were 302.5 ± 38.1 and 251.5 ± 38.7 for the younger and older groups, respectively (*p* < 0.05) (Table 1).

## 3. Discussion

In this study, we investigated the protein composition of human FF obtained from females undergoing ICSI using the MALDI-TOF-TOF technique. This is the first report comparing protein expression in the FF between older and younger women and identified five differentially-expressed proteins in the FFs of the two groups. Based on previous studies in ART, the success rate declines with age. There is a lack of assessment of oocyte quality or ability to predict the success of IVF treatment [46]. Because clinical outcomes of IVF are highly dependent on age, we sought to identify the differences in the total number of retrieved mature oocytes. Compared with the younger group, women in the older group had a significantly lower number of oocytes. Certain components of FF might be used as indicators for the maturation and the quantity of the oocytes. Proteins can be used as biomarkers for reproductive diseases using both FF and plasma.

The majority of all proteins identified in this study were plasma proteins. This can be explained with the diffusion of plasma proteins over the blood-follicle barrier, which increases in its permeability during follicle maturation [2].

On the basis of the proteome analysis presented above, components of the complement cascade (components C3, C4) were found more abundant in the younger group compared to the older group. The involvement of the complement cascade in the innate immunity response in human FF was established [46].

Gonzales *et al.* (1992) found significantly higher concentrations of the C3 complement fraction in FF from oocytes that had been fertilized in comparison with those non-fertilized, which confirms our study [47].

Jarkovska *et al.* (2010) indicated the low levels of components complement C3 and C4 together with the high level of C9 in FF compared to the plasma. They demonstrated that complement activation causes the deficiency of vascular endothelial growth factor (VEGF), the growth factor that is consequently required for oocyte maturation [46].

In contrast to our study, Estes *et al.* (2009) assessed the FF of IVF patients to identify biomarkers to predict IVF success. They showed decreased expression of complement C3 in the successful group [41]. Although, our results showed a reduction of components C3 and C4 in the FF of the aged group, additional studies are needed to establish the actual influence of the complement cascade on reproductive aging and IVF outcome.

Some of the proteins contributing to the concept of “ageing” in the FF proteome are known to be involved in iron transport, such as hemopexin and serotransferrin (STF).

In this regard, our results also showed that two proteins, hemopexin precursor (spots 75, 86, 88, 90, 91, 181, 182) and STF (spots 108, 111, 112, 113, 261, 254), decreased significantly in the older group. STF is a monomer composed of two homologous domains known as a circulating iron carrier protein, which is produced in the testis [48], the ovary and FF [41,49,50]. STF acts as an iron chelator to dampen down the generation of reactive oxygen species, which may promote atresia in the follicle [51]. The expression of transferrin has been demonstrated in granulosa cells in human [52] and mouse follicles [50].

Angelucci *et al.* (2006) and Jarkovska *et al.* (2010) found significantly higher concentrations of transferrin in FF compared to the plasma. A higher concentration of transferrin in FF could be the result of the local synthesis of the protein by granulosa cells [38,46]. Spitzer *et al.* (1996) compared the protein patterns originating from the fluids of mature and immature human follicles and showed significantly higher concentrations of transferrin in FF that contains fertilized oocytes [35].

In humans, transferrin concentrations in FF are highly related to the circulating level, the degree of follicular maturity and steroidogenesis [52,53,54]. In our study, the serum transferrin level was significantly higher in the younger group; thus, transferrin was chosen as a serum protein, which might reflect reproductive aging and ovarian activity. It seems that transferrin secretion would be influenced by aging and would lead to lower numbers of metaphase II oocytes. In accordance with our study and the studies mentioned above, STF could be considered as a potential biomarker for folliculogenesis, oocyte maturation and reproductive aging. However, further research is necessary to elucidate the precision of this idea and to verify the role of transferrin in oocyte and embryo quality.

Our results showed that hemopexin decreased in the FF of the aged group. Free heme is a potential source of iron that is toxic for cells and catalyzes the formation of free radicals. Hemopexin binds free heme and mediates its uptake in liver cells [55]. Plasma hemopexin promotes the metabolic processing of heme and inhibits the toxicity resulting from the oxidative catalytic activity of heme. As well, the heme-hemopexin complex can activate the signaling pathways, subsequently promoting cell survival and modulating gene expression [56]. Hemopexin can act as an extracellular antioxidant against hemoglobin-mediated damage in inflammatory states [57]. Repeated exposure of oocytes and granulosa cells to oxidative stress could be associated with reproductive failure. Oxidative stress might be responsible, at least in part, for the reduced reproductive potential related to ageing. Thus hemopexin might be involved in folliculogenesis and could be used as a biomarker of ovarian aging. Local synthesis of hemopexin in granulosa cells and defense against oxidative stress should be well documented by further studies.

The other finding of this study was the downregulation of kininogen in the aged group. It is clear that kallikrein activity and kininogen levels increase in the ovary, preceding and at the time of ovulation [58,59,60]. The plasma kinin system has an important role in various physiological processes, such as ovulation [58], and has been found in the ovary and FF [46,49]. Kininogen would be proangiogenic and stimulate angiogenesis [61]. It has been reported that angiogenesis is suppressed by kininogen deficiency in rats [62]. Physiological angiogenesis in the ovarian follicles and corpus luteum is essential for follicular growth, oocyte quality and mammalian reproduction. Failures in ovarian angiogenesis may be the reason for several ovarian dysfunctions [63,64]. Tatone *et al.* (2008) suggested that age-related nuclear and cytoplasmic damage may occur as a result of inadequate ovarian angiogenesis in primordial follicles, as well as in ovarian stroma vessels [65], which confirms our results. Taken together, it seems that decrease of kininogen in the older group may be correlated to angiogenesis deficiency and, subsequently, poor oocyte quality and number. Indeed kininogen could act as another biomarker in reproductive aging.

## 4. Experimental Section

### 4.1. Patients

We recruited healthy, ovulatory women aged 38–42 years (*n* = 12) and 20–32 years (*n* = 12) with male factor infertility undergoing ICSI and enrolled them [65]. Inclusion criteria were basal follicle stimulating hormone (FSH) concentration <10 mIU/mL on the third day of the cycle, body mass index (BMI) (range: 15–29.3) and a single etiology for ART (only male factor). Patients with known polycystic ovarian syndrome, endometriosis and tubal disease with hydrosalpinx on transvaginal ultrasonography were excluded. No patients in either group had a medical history of depression, diabetes, eating disorder, cancer, hypertension, hyperthyroidism or fibroid uterus. The characteristics of patients did not differ significantly between groups, except for age [32,66]. Informed consent was obtained from each couple. This study was approved by the Ethics Committee of Ahvaz Jundishapour University of Medical Sciences.

### 4.2. Ovarian Stimulation, FF Sampling and Oocyte Collection

All patients underwent controlled ovarian hyperstimulation (COH) according to established protocols. When at least three follicles reached ≥18 mm, a 10,000 IU of human chorionic gonadotropin (hCG) (Choriomon, IBSA, Lugano, Switzerland) was administered intramuscularly 34–38 h after hCG injection, under ultrasound guidance; follicles larger than 15 mm in diameter were aspirated with a 17-gauge Cook needle, and oocytes were retrieved [23,67,68]. After oocyte isolation, FF from 3 mature follicles (≥18 mm) was pooled and centrifuged at 13,000× *g* for 10 min to remove cells and insoluble particles. Then, the supernatant was transferred to sterile cryovial and stored at −80 °C for further study. Specimens with blood contamination were discarded [24,41].

### 4.3. Assessment of Oocytes Maturation (Metaphase II Oocytes) and Fertilization

Nuclear maturation of oocytes was determined by the identification of the first polar body. Oocytes were assessed for maturity and underwent ICSI. The following day, normal fertilization was confirmed by the presence of two pronuclei (2PN) and two polar bodies. Data on the number of mature oocytes was recorded.

### 4.4. Determination of FSH Concentrations in Serum

Blood samples were obtained during the early follicular phase (Days 3–5) from the patients, and the follicle stimulating hormone (FSH) concentration was measured using an ELISA method.

### 4.5. Sample Preparation and Two-Dimensional Gel Electrophoresis

#### 4.5.1. Depletion of Albumin/IgG from FF Using Qproteome Albumin/IgG Depletion Plates

Albumin and IgG were removed from FF using Qproteome Albumin/IgG Depletion Plates (QIAGEN, Cat. No. 37009), according to the procedures recommended by the manufacturer instructions.

#### 4.5.2. Protein Assay

The total protein concentration of crude and depleted FF was measured by the Bradford method with bovine albumin as the standard. Each sample was analyzed individually in triplicates [69].

### 4.6. Two-Dimensional Gel Electrophoresis

Two hundred fifty micrograms and 1000 μg of albumin/IgG-depleted protein FF (for the analytical and the preparative gels, respectively) were dissolved in rehydration solution containing 8 M urea, 2 M thiourea, 20 mM DTT, 4% CHAPS (3-cholamido-propyl)di-methylammonio]-1-propanesulfonate) (*w*/*v*), 2% (*v*/*v*), IPG buffer (PH 3–10), 5% *v*/*v* protease inhibitor cocktail and a trace of bromophenol blue for isoelectric focusing (IEF), then centrifuged at 14,000× *g* for 20 min [24,38,70]. IEF was performed in IPG strips (pH 3–10, non-linear) using an IPGphor IEF system (Amersham pharmacia Biotech, Uppsala, Sweden) and the following program: 500 V for 1 h (step and hold), 1000 V for 1 h (gradient), 8000 V for 2 h (gradient) and 8000 V for 8 h (step and hold) for a total of 42,000 Vh [24,38,71]. After the IEF, the IPG strips were equilibrated in the equilibration solution (50 mM Tris-HCl (pH 8.8), containing 2% (*w*/*v*) SDS, 1% (*w*/*v*) dithiothreitol (DTT), 6 M urea and 30% (*w*/*v*) glycerol for 15 min and then in the same solution containing 5% iodoacetamide instead of DTT. The second dimension was carried out in 13.5% separating gels using the Dodeca Cell System (Bio-Rad Laboratories, Hercules CA, USA) at a constant voltage and 50 mA per gel at 15 °C for 7 h [38,70,72].

### 4.7. Silver and Coomassie Blue Staining

After the electrophoresis step, analytical gels were stained with acidic silver nitrate (a very sensitive tool for protein visualization) [70,73], while preparative gels used for matrix-assisted laser desorption-ionization time-of-flight/time-of-flight (MALDI-TOF-TOF) protein identification were stained with Coomassie blue R-350 (PlusOne Coomassie Tablets, PhastGel Blue, GE Healthcare), which is more sensitive than the commercially available R-150 and R-250 dyes. For silver staining, the gel was fixed in the appropriate first fixation solution (40% methanol and 7% acetic acid) and the second fixation solution (5% methanol and 7% acetic acid) for 30 min with shacking, respectively. The gel was submerged in 10% glutaraldehyde for 30 min followed by washes in deionized water, then placed in deionized water containing DTT (5 µg/µL) for 30 min and in 1% silver nitrate for 30 min with gentle shacking and rinsed briefly in deionized water. This was placed and developed in developing solution (3% *w*/*v* sodium carbonate and 0.019% *w*/*v* formaldehyde). After the detection of protein spots, the gel was incubated in the stop solution (second fixation solution) for 30 min and finally stored in 7% acetic acid for future analysis [70]. For Coomassie blue staining, we used 200 mL of a 0.1% staining solution [74]. Triplicate gels were run for each sample.

### 4.8. Imaging and Statistical Analysis

The gels were scanned with the Image scanner (Amersham Pharmacia, Piscataway, NJ, USA), saved as TIFF images and analyzed with Image Master 2D Platinum 6.0 software (GE Healthcare) for spot detection, quantification, comparative and statistical analysis. Then, manual editing was performed, and the results were in agreement with the visual inspection. The volume of each spot from three replicate gels was normalized against total spot volume, quantiﬁed and subjected to the Student’s *t-*test (*p* < 0.05). Only those spots that were present on all three replicate gels were quantiﬁed and enrolled for statistical analysis. The spots with significant differences in expression levels (*p* < 0.05) between the two groups were considered up- or down-regulated. The ratios of the volume value of older *versus* younger groups were calculated and listed as the “fold change” column in Table 2. A fold change of 1.5 was then chosen as the threshold of expression variation. The differentially-expressed protein spots were selected as candidate proteins for MALDI-TOF-TOF analysis.

### 4.9. Protein Identification by Mass Spectrometry and Sequence Database Searching

#### 4.9.1. MALDI-TOF-TOF MS

Differentially-expressed spots were manually excised from preparative Coomassie blue-stained gels. Analysis was carried out by the Proteomics Laboratory; University of York, U.K., using MALDI-TOF-TOF mass spectrometry. Gel pieces were washed twice with 50% (*v*:**v**) aqueous acetonitrile containing 25 mM ammonium bicarbonate, then once with acetonitrile and dried in a vacuum concentrator for 20 min. Proteins in the gel pieces were digested using a trypsin solution containing 12 ng/lL (10 lL) trypsin in 50 mM ammonium bicarbonate solution for 45 min at 4 °C.

Sequencing-grade, modified porcine trypsin (Promega) was dissolved in the 50 mM acetic acid supplied by the manufacturer, then diluted five-fold by adding 25 mM ammonium bicarbonate to give a final trypsin concentration of 0.02 mg/mL. Gel pieces were rehydrated by adding 10 mL of trypsin solution, and after 30 min, enough 25 mM ammonium bicarbonate solution was added to cover the gel pieces. Digests were incubated overnight at 37 °C. A 1-mL aliquot of each peptide mixture was applied directly to the ground steel MALDI target plate, followed immediately by an equal volume of a freshly-prepared 5 mg/mL solution of 4-hydroxy-a-cyano-cinnamic acid (Sigma) in 50% aqueous (*v*:*v*) acetonitrile containing 0.1%, trifluoroacetic acid (*v*:*v*). Positive-ion MALDI mass spectra were obtained using a Bruker ultraflex III in reflectron mode, equipped with a Nd:YAG smart beam laser. MS spectra were acquired over a mass range of *m*/*z* 800–4000 °C. Final mass spectra were externally calibrated against an adjacent spot containing 6 peptides (des-Arg^1^-bradykinin, 904.681; angiotensin I, 1296.685; Glu^1^-fibrinopeptide B, 1750.677; ACTH (Adrenocorticotropic Hormone) (1–17 clip), 2093.086; ACTH (18–39 clip), 2465.198; ACTH (7–38 clip), 3657.929.). Monoisotopic masses were obtained using a SNAP (Sophisticated Numerical Annotation Procedure) averaging algorithm (C 4.9384, N 1.3577, O 1.4773, S 0.0417, H 7.7583) and an S/N threshold of 2. For each spot, the ten strongest peaks of interest, with an S/N greater than 30, were selected for MS/MS fragmentation. Fragmentation was performed in LIFT mode without the introduction of a collision gas. The default calibration was used for MS/MS spectra, which were baseline-subtracted and smoothed (Savitzky–Golay; width: 0.15 *m*/*z*; cycles: 4); monoisotopic peak detection used a SNAP averaging algorithm (C 4.9384, N 1.3577, O 1.4773, S 0.0417, H 7.7583) with a minimum S/N of 6. Bruker flexAnalysis software (version 3.3) was used to perform the spectral processing and peak list generation for both the MS and MS/MS spectra.

#### 4.9.2. Data Processing and Protein Identification

Tandem mass spectral data were submitted to database searching using a locally-running copy of the MASCOT program against the IPI (International Protein Index) human database (Matrix Science Ltd., version 2.1), through the Bruker ProteinScape interface (version 2.1). The following specified parameters were applied for the database search: database (SwissProt); taxonomy (*Homo sapiens*); proteolytic enzyme (trypsin); peptide mass tolerance (±100 ppm); fragment mass tolerance (±0.5 Da); fixed modification (carbamidomethyl (Cys)); variable modification (oxidation (Met)); and max missed cleavage (1).

#### 4.9.3. Determination of Transferrin Concentrations in Serum

Serum transferrin was measured according to the manufacturers’ instructions. Immunoturbidimetry was performed using the transferrin test kit (Parsazmun, Iran) containing anti-human transferrin goat serum and polyethylene glycol to enhance the immunoprecipitation.

### 4.10. Statistical Analysis

All data were presented as the mean ± standard deviation. The Student’s *t*-test was used for statistical comparisons. The statistically significant level was *p* < 0.05.

## 5. Conclusions

We evaluated the protein expression of FF with the goal of detecting novel candidate markers for oocyte number and, especially, reproductive aging in the IVF cycle. The results indicated that transferrin, kininogen and hemopexin may play important roles in folliculogenesis. To our knowledge, no studies have been published about the association between these proteins detected by MS in our study and ovarian aging; thus, this needs additional investigation.

Because the precise function of these proteins in the ovary is not known properly, further studies, including protein characterization, specific antibody generation and immune assay development, are required to understand the function of these markers in female fertility. Additionally, it would also be important to research the translation of these protein markers with respect to serum testing. This could be useful before the initiation of any IVF cycles.

In conclusion, we have observed the variable expression of certain proteins. Further studies are needed to clarify the exact mechanism of action of these proteins. Further investigation may elucidate proteins in the FF and serum of older women and possibly lead to the identification of markers of poor oocyte and embryo quality, as well as potential therapeutic targets for improving reproductive success following ART, particularly in older women
